# Microclimatic effects on activity patterns in a montane chimpanzee population

**DOI:** 10.1098/rsos.261020

**Published:** 2026-10-14

**Authors:** Hassan Al-Razi, Fidele Muhayeyezu, Felix Mulindahabi, Protais Niyigaba, Drew Arthur Bantlin, Beth A. Kaplin, Shane K. Maloney, Cyril C. Grueter

**Affiliations:** ^1^ Department of Anatomy, Physiology and Human Biology, School of Human Sciences, The University of Western Australia, Perth, WA 6009, Australia; ^2^ Centre for Evolutionary Biology, School of Biological Sciences, The University of Western Australia, Perth, WA 6009, Australia; ^3^ Centre of Excellence in Biodiversity and Natural Resource Management, University of Rwanda, Kigali, Rwanda; ^4^ Nyungwe Management Company, African Parks, Kigali, Rwanda; ^5^ School for the Environment, University of Massachusetts, Boston, MA, USA; ^6^ Institute of Human Sciences, School of Anthropology and Museum Ethnography, University of Oxford, Oxford, UK; ^7^ International Centre for Biodiversity and Primate Conservation, Dali University, Dali, Yunnan, People's Republic of China

**Keywords:** activity rhythms, adaptation, behavioural thermoregulation, great ape, Nyungwe National Park

## Abstract

Climatic variation can shape primate activity budgets by altering the energetic and thermoregulatory costs of feeding, resting, locomotion and social behaviour. However, little is known about how chimpanzees that inhabit cool, wet montane forests adjust their activity in response to fine-scale meteorological conditions. We examined the effects of temperature, absolute humidity (water vapour pressure (WVP)), wind speed and rainfall on eastern chimpanzees (*Pan troglodytes schweinfurthii*) in Nyungwe National Park, Rwanda, one of the highest-elevation chimpanzee habitats. From March 2024 to February 2025, we collected 1933 party scans and 5661 individual scans, together with scan-level microclimatic measurements. Rainfall showed the clearest behavioural association: feeding probability declined during rainfall, whereas resting increased. Wind speed was negatively associated with both locomotion and grooming, suggesting that windier conditions may constrain movement and social behaviour. Temperature, WVP and fruit availability showed no clear association with any of the four behaviours. Feeding probability was higher and resting probability lower in the wet season than in the dry season. Harmonic beta-binomial generalized linear mixed-effects models revealed pronounced daily rhythmicity in feeding and resting. Overall, Nyungwe chimpanzees maintained broadly conserved daily activity rhythms while selectively adjusting behavioural allocation in response to immediate meteorological conditions.

## 1. Introduction

Climatic factors, particularly temperature, humidity, wind speed, rainfall and solar radiation, are known to influence the ecology of animals and can shape population density, habitat selection, group size and reproductive success [1–7]. Climatic conditions can also directly affect the activity budget of an animal by constraining energetic expenditure and daily behavioural organization [8–10]. Although other factors, such as food availability, mate availability and predation pressure, also influence activity patterns, these factors are themselves directly or indirectly shaped by climatic conditions [11–16]. There is evidence that homeothermic animals, including primates, can alter their behavioural strategies in response to climatic factors in ways that improve heat balance and reduce the energetic and water costs of thermoregulation [2,9,17,18]. Such behavioural thermoregulation operates by modifying heat exchanges with the surrounding environment through radiation, convection and conduction [19]. These exchanges are shaped by ambient temperature, relative humidity, solar radiation, rainfall and wind speed, which together define an animal's thermal environment [20]. Consequently, animals employ a suite of thermal behaviours, including modifying activity and rest patterns, adopting thermally efficient postures and selecting microhabitats or resting sites that facilitate heat conservation, heat gain or heat dissipation under varying environmental conditions [21–24].

Although most primates inhabit tropical and subtropical regions, they experience substantial climatic variation across their geographic ranges [2,25,26]. Within these regions, species occupy habitats that range from lowland forests to montane environments. Some persist in extremely hot and arid habitats; for example, the hamadryas baboon (*Papio hamadryas*) inhabits arid regions of Africa where daytime temperatures can exceed 38°C [27,28]. By contrast, other primates inhabit cooler montane and temperate settings where lower temperatures and variation in humidity markedly alter the thermal conditions and overall thermal stress. A small number extend into cold climates where temperatures can regularly fall below 0°C [29,30]. Across this broad thermal spectrum, activity-budget adjustment is one of the most widely reported behavioural responses to thermal stress in primates [19,31–38]. These strategies are often combined, particularly through modifications of activity budgets in response to thermal constraints. In hot environments, primates typically reduce energetically costly behaviours that also generate metabolic heat, such as feeding and locomotion, while increasing the time that they spend resting to limit the production of metabolic heat and thus reduce thermal load. It might seem counter-intuitive that in cooler or wetter environments, low temperatures and rainfall are also frequently associated with reduced feeding and locomotion and increased resting [7,16,39–44], but at low temperature the increase in convective heat loss encountered during feeding and locomotion presumably is larger than the extra metabolic heat that is generated by those activities. However, some primates that inhabit extremely cold environments, such as Tibetan macaques (*Macaca thibetana*), black-and-white snub-nosed monkeys (*Rhinopithecus bieti*) and golden snub-nosed monkeys (*Rhinopithecus roxellana*), increase their feeding effort under cooler conditions [11,29,45]. This pattern may reflect the complex relationships that exist in an animal's energy balance, because the generation of metabolic heat, whether that be by shivering or activity, requires an increase in food intake or the mobilization of body energy stores. However, at least some of the extra feeding activity could arise from seasonal shifts towards lower-quality diets rather than increased energetic expenditure *per se* [46]. By contrast, relative humidity has generally been shown to have weak or negligible effects on primate activity budgets in cooler environments [7,40].

Chimpanzees (*Pan troglodytes*) have a wider geographic distribution than any other great ape and occupy a broad range of habitat types, from dry savannah mosaics to moist montane forests [47]. As a result of this habitat diversity, chimpanzee populations can be exposed to markedly different climatic conditions. While some populations inhabit regions that are characterized by extreme heat, others live in cooler, high-elevation forests where low temperatures and high rainfall prevail [48–52]. To cope with this climatic heterogeneity, rather than rely on autonomic thermoregulatory mechanisms that can incur large costs in terms of energy or water use, chimpanzees rely primarily on behavioural thermoregulation and adjust their activity budgets, microhabitat use and nesting behaviour in ways that help them to maintain thermal balance [37,52–54]. For example, during periods of high ambient temperature, chimpanzees adjust their activity by resting more and feeding less [54]. Chimpanzees also employ microhabitat selection during hot conditions as a thermoregulatory strategy, preferentially using shaded forest interiors, dense vegetation or riparian areas [37]. By contrast, during cooler periods, they use more exposed or sunlit locations [51,54–56]. Nesting behaviour further contributes to thermal regulation, with adjustments in nest height, site selection and structural properties in relation to temperature, rainfall and wind exposure, including the construction of more insulated nests when it is cold or wet [53,57–59].

Several studies have examined the effect of climatic factors on activity patterns across different primate species. Some have focused on macroclimatic variation, such as seasonal changes in temperature and rainfall [56,60–63], whereas others have investigated microclimatic influences by analysing fine-scale daily variation in weather conditions using field-based measurements or data from nearby meteorologic al stations [6,20,35,36,40,64–69]. For chimpanzees, the number of studies that have examined the effect of climatic factors on behaviour remains relatively limited, and most have focused on seasonal variation [54–56,62]. Research has therefore been concentrated primarily on populations that inhabit hot, open or savannah-mosaic environments, where thermal challenges are driven by high ambient temperatures and solar radiation. Consequently, most empirical studies have examined behavioural responses to elevated ambient temperatures [52,54–56,58]. Little is known about how chimpanzees cope with chronic exposure to cool, wet and thermally variable environments, particularly at high elevation. To date, no study has explicitly examined behavioural thermoregulation in a montane forest occupied by chimpanzees, such as Nyungwe National Park (NNP), which represents one of the highest-elevation habitats of chimpanzees, at approximately 1600–2950 m.a.s.l. The park is characterized by persistently low temperatures, high rainfall and frequent wind exposure [47,70–72], creating a markedly different thermal environment from the hot, open habitats where most previous studies have been conducted. In such environments, ambient temperature can fall below that generally inferred to be thermally neutral for chimpanzees, approximately 17–29°C [73], potentially imposing sustained cold stress rather than episodic heat stress. The lack of data on chimpanzees in cold conditions represents a major gap in our understanding of chimpanzee thermal ecology. Moreover, how such climatic conditions shape the diel organization of activity remains largely unexplored. In particular, the timing and structure of feeding and resting rhythms, activities that dominate chimpanzee activity budgets and are closely linked to energetic and thermoregulatory trade-offs, remain poorly understood in cool, high-elevation forests, especially in relation to patterns described for savannah populations.

Changes in activity budgets can influence thermoregulation because different behaviours have distinct consequences for metabolic heat production, heat loss and energy balance. Locomotion is energetically costly and increases metabolic heat production, whereas feeding involves both energetic expenditure during foraging and energy acquisition through food intake. The thermal consequences of these behaviours, therefore, depend on the surrounding microclimatic conditions [2,18,74,75]. In cool environments, low ambient temperature can increase the gradient for heat loss between the body and the surrounding air, while wind can further increase convective heat loss by removing the insulating boundary layer around the body surface [17–20]. Rainfall may add further costs by wetting the pelage and reducing its insulating capacity, while also constraining movement or foraging under wet conditions [39,40,76]. Under such conditions, primates may reduce energetically costly activities and increase resting when the energetic and thermoregulatory costs of activity exceed the benefits of immediate foraging or movement [2,9,40,77]. Because activity can contribute to heat production, but feeding is necessary to maintain energy balance, climatic effects on activity budgets are unlikely to follow a single direction across all conditions. Instead, adjustment to an activity budget reflects a flexible trade-off between energy gain, energy expenditure and thermoregulatory costs [2,8,18,77]. We examined the effects of temperature, humidity, rainfall and wind speed on the activity of eastern chimpanzees (*Pan troglodytes schweinfurthii*) in NNP to test whether chimpanzees that live in a cool montane forest adjust their activity budget in response to short-term microclimatic variation. By integrating fine-scale microclimatic measurements with detailed behavioural observations, we developed four predictions: (i) under lower temperatures and during rainfall, chimpanzees would reduce feeding and increase resting because cold and wet conditions may increase heat loss and make feeding more difficult; (ii) because wind can exacerbate convective heat loss in a montane environment and reduce branch stability in the canopy, we predicted that increasing wind speed would be associated with reduced feeding and locomotion and increased resting; (iii) although humidity declines seasonally during Nyungwe's long dry season, the forest is generally characterized by a humid montane climate and so we predicted that humidity would have little effect on chimpanzee activity allocation; and finally (iv) we predicted that Nyungwe chimpanzees, like other primates and chimpanzee populations elsewhere, would show clear daily rhythmicity in feeding and resting, reflecting the need to maintain energetic balance in a cool montane environment.

## 2. Methods

### 2.1. Study area and species

NNP lies in the southwest of Rwanda (2°17′–2°50′ S, 29°07′–29°26′ E) and covers roughly 1013 km² of tropical montane forest along the Albertine Rift. The park extends across an altitudinal gradient from approximately 1600 to 2950 m, creating a cool and consistently moist climatic environment. Although the onset and intensity of the two wet seasons each year are highly variable, there is generally a major wet season from March to May, a major dry season from June to August, and a short dry season from December to January [72,78–80].

The forest forms part of the wider Nyungwe–Kibira landscape, one of the most extensive continuous montane forests that remains in Africa [81]. Nyungwe supports exceptional biodiversity, including more than 80 mammal species, 25 Albertine Rift–endemic birds and 12 non-human primate species [81]. The park is home to Rwanda's largest chimpanzee population, estimated at approximately 380 individuals [82]. Vegetation is a heterogeneous mosaic of primary and secondary forest, bamboo stands, swamp systems and open montane grasslands, with local variation influenced by canopy openness and moisture availability.

We studied the Mayebe chimpanzee community, a well-habituated group of approximately 67 identified individuals. At the time of the study, the community comprised 14 adult males, four subadult males, 18 adult females, seven subadult females, 12 juveniles and 12 infants, with an additional 11 individuals that remained unidentified. The Mayebe community ranges between Uwinka and Mount Bigugu within the NNP [70]. Depending on the method that is used to estimate the size of their home range, it spans approximately 38–48 km² [70] and includes the highest known altitudinal limit recorded for chimpanzees, reaching 2950 m.a.s.l. The community shows a typical multi-male–multi-female social organization and follows a fission–fusion system in which party size and composition change frequently [83].

### 2.2. Data collection

#### 2.2.1. Behavioural data

With the assistance of two trained field assistants, we collected behavioural data from March 2024 to February 2025 on approximately 8 days each month. Using instantaneous scan sampling [84], we recorded 1933 systematic party scans and 5661 individual scans, totalling 486 contact hours. Because chimpanzees live in fission–fusion societies in which community members form temporary parties that vary in size and composition, we defined a party as all of the individuals that were visible to the observers and observed in spatial association, moving, feeding or resting together during a scan period [85,86]. Owing to the challenging terrain, dense vegetation and the species’ extensive home range, individual focal follows were difficult; instead, we used the scan-sampling protocol following Matthews *et al.* [87]. During each scan, individuals were recorded following a fixed left-to-right sequence to ensure unbiased sampling, prevent resampling of the same individual within a scan period, and to minimize preferential attention to conspicuous behaviours [88–90]. We began observations before dawn by locating the previous night's nesting sites, and we followed the chimpanzees throughout the day until they constructed their night nests, yielding full-day party-level behavioural data. Alternatively, we located chimpanzees by following the direction of vocalizations, fresh tracks and recently used feeding sites. We followed each chimpanzee party for as long as possible (field effort exceeded 8 h on 49 of 89 observation days), with a median daily contact time of 5.5 h (range: 1.0–9.5 h). If a party was lost, we attempted to relocate it; if unsuccessful, we continued data collection on the next party encountered [91].

To test the effects of meteorological variables on the activity of chimpanzees, we categorized chimpanzee behaviour into four main activities: feeding, resting, locomotion, and grooming [54,92]. We excluded other social behaviours, such as play and aggression, from the analyses because of the very low number of observations of those behaviours and the difficulty of reliably assessing relationships between a small number of observations and climatic variables. Feeding was defined as all food-processing and ingestion behaviours; resting encompassed any inactive state irrespective of posture (e.g. sitting, lying or stationary standing); locomotion included walking, climbing and other positional movements; and grooming comprised both self-grooming and allogrooming. Individuals were classified into five age–sex categories (adult male, adult female, subadult male, subadult female and juvenile) following established criteria for eastern chimpanzees [92]. Although age–sex class was recorded during scan sampling, individual identity was not a focus of the sampling design or analysis. Age–sex class was therefore not included in the scan-level models.

#### 2.2.2. Weather data

To investigate daily and monthly variation in meteorological conditions, we recorded temperature, relative humidity, wind speed and rainfall using an automated mini weather station (WS1081 Ver3 TESA Professional Weather Station). Temperature, relative humidity and wind speed were recorded at 30-minute intervals, whereas rainfall was recorded as an hourly total. The station was permanently installed in an open area at the research camp, at an elevation of approximately 2200 m.a.s.l. For the descriptive daytime summary, we restricted the station data to records collected between 06.00 and 18.00 local time. Daily mean temperature, relative humidity, water vapour pressure (WVP) and wind speed were calculated from these sub-daily records. Each hourly rainfall value appeared identically in the two corresponding 30-minute records in the downloaded dataset; therefore, one of the two identical values within each hour was removed during data cleaning, retaining a single hourly rainfall value. Daily daytime rainfall was then calculated by summing the retained hourly values within the daytime window. For each month, we reported the minimum and maximum individual daytime measurements of temperature, relative humidity, WVP and wind speed, together with the minimum and maximum daily daytime rainfall totals. Monthly variation in daily minimum and maximum temperature and WVP was summarized using box plots. Diel profiles of daytime temperature and WVP were generated by averaging observations across days within each month for each 30-minute time-of-day interval and plotting the resulting monthly mean curves.

To measure the effects of meteorological factors on different behaviours, during the observations on the chimpanzees, we recorded fine-scale microclimatic data on ambient temperature, relative humidity, wind speed and rainfall at each scan. Rainfall was recorded as a binary variable (present/absent). During each behavioural scan, wind speed and ambient temperature were measured using a handheld anemometer (Extech 45158 Rotary Vane), while relative humidity was measured using a handheld heat-stress meter (Extech HT30 Heat Stress WBGT Meter). Microclimatic measurements were taken at positions that matched the immediate exposure condition of the chimpanzees. When the majority of the individuals were in direct sunlight, temperature and relative humidity were recorded in a sun-exposed location; when they were in shade, measurements were taken in a shaded location. To obtain a wind speed estimate that more closely approximated the exposure of arboreal individuals, we attempted to reduce the vertical difference between the chimpanzees and the measurement location by having a field assistant climb the nearest accessible slope or elevated point. The anemometer was held facing the prevailing wind direction and away from obstructions to minimize turbulence. Scan-level wind speed ranged from 0 to 1.7 m s^−1^, with a median of 0 m s^−1^; no detectable wind was recorded during 59% of scans.

Relative humidity is strongly temperature-dependent; therefore, for analysis, we converted relative humidity to WVP. WVP provides a measure of absolute atmospheric humidity that is more suitable to evaluate humidity effects alongside temperature. Following Barenbrug [93], Tetens [94] and Buck [95], we calculated WVP as

$$vapour\;pressure\;of\;water\left(WVP\right)=0.6105\times \exp\left[\frac{17.27\;\times \;T_{a}}{\left(237.3\;+\;T_{a}\right)}\right]\times \frac{RH}{100},$$

where:

WVP = water vapour pressure (kPa),

T_a_ = air temperature (°C),

RH = relative humidity (%), and

exp = exponential function (eˣ).

#### 2.2.3. Fruit availability index

The monthly fruit availability index (FAI) was calculated using fruit-phenology data obtained through regular monthly monitoring of key fruiting plant species within the study area. From this dataset, we selected 19 tree species identified as important food resources for chimpanzees during the study. Within the chimpanzees’ home range, we monitored 620 individual trees along 17 selected trails, with each species represented by more than 15 individuals. We included only trees with a diameter at breast height (DBH) of at least 10 cm and crowns that were clearly visible from the trail, thereby improving the reliability of the monthly fruiting assessments. For each tree, we scored the proportion of the crown bearing fruit using a five-point ordinal scale: 0 (less than 1%), 1 (1–25%), 2 (26–50%), 3 (51–75%) and 4 (76–100%).

To account for variation in tree size, DBH was converted from centimetres to metres, and the basal area of each tree was calculated as

$$\begin{array}{r} BA_{i}=\frac{\pi }{4}{\left(\frac{DBH_{i,cm}}{100}\right)}^{2}. \end{array}$$

We then calculated the monthly fruit availability index by multiplying each tree's fruit score by its basal area and summing these values across all trees with valid observations in that month,

$$\begin{array}{r} FAI_{m}=\overset{N_{m}}{\underset{i=1}{\sum }}\left(FruitScore_{i,m}\times BA_{i}\right), \end{array}$$

where FAI*_m_* is the fruit availability index for month *m*, FruitScore*_i_,_m_* is the fruit score of tree *i* in month *m*, BA*_i_* is the basal area of tree *i*, expressed in square metres and *N_m_* is the number of monitored trees with valid fruiting observations in month *m*.

### 2.3. Statistical analysis

Using R v. 4.3.0 [96], we examined associations between activity and fine-scale weather using generalized linear mixed models (GLMMs) fitted with the glmmTMB package [97]. We analysed feeding, resting, locomotion and grooming separately at the scan level because individuals within a scan shared the same sampling occasion and climatic measurements. We created a unique scan identifier by combining observation date and ScanID. For each behaviour, we modelled the number of individuals that were performing the focal behaviour relative to the number that were performing all other behaviours in the same scan. We fitted beta-binomial GLMMs with a logit link to account for variation in scan-level sample size and extra-binomial variation in behavioural proportions. Because the four behaviours were mutually exclusive components of the activity budget, we interpreted the models as separate one-versus-rest analyses rather than as independent components of a jointly modelled composition.

We included temperature, WVP, wind speed, rainfall, monthly FAI, time of day and season as fixed effects. Based on observation time, scans were classified as morning (06.00–10.59), midday (11.00–14.59) or afternoon (15.00–18.59). We excluded 39 scans that were conducted before 06.00 because they fell outside these time-of-day categories. Rainfall was modelled as a binary variable, and FAI was included to account for monthly variation in fruit availability. Each behavioural scan was assigned the FAI value that corresponded to its month of observation. Because phenological monitoring was conducted monthly, FAI comprised 12 unique values across the study period, and inference for this predictor was therefore based on temporal variation among months. We did not include age–sex class because the response variable represented the overall proportion of individuals performing each behaviour within a scan, rather than repeated observations of identified individuals. Observation date was included as a random intercept to account for repeated scans within days. We standardized continuous predictors to a mean of zero and a standard deviation of one at the scan level and assessed multi-collinearity using variance inflation factors (VIFs) [98]. The VIF values for all variables were less than 3. We evaluated model terms with type II Wald χ² tests and evaluated model adequacy using simulation-based residual diagnostics in DHARMa [99]. The full model for each behaviour was

$$\begin{array}{rcl} cbind\left(n_{focal},n_{other}\right)∼ & & temperature+WVP+wind+rainfall+FAI+time\;of\;day\\ & & +season+\left(1|date\right). \end{array}$$

To test whether fine-scale weather collectively improved model fit, we compared each full model with a control-only model that contained FAI, time of day, season and the random intercept for date. We used likelihood-ratio tests and Akaike's information criterion (AIC) for these comparisons. The likelihood-ratio test, therefore, assessed whether temperature, WVP, wind speed and rainfall explained additional variation beyond the control variables.

We modelled daytime variation in feeding and resting using harmonic beta-binomial GLMMs implemented in glmmTMB [97]. Models were fitted at the scan level. For each behaviour, the response comprised the number of individuals that were performing the focal behaviour relative to the number that were performing all other behaviours within the same scan. We used a beta-binomial distribution with a logit link to account for variation in scan size and extra-binomial variation in behavioural proportions. Time of day was represented using first- and second-order sine and cosine terms,

$$\begin{array}{r} cbind\left(n_{focal},n_{other}\right)∼sin1\;+\;cos1\;+\;sin2\;+\;cos2\;+\left(1|Date\right)\;. \end{array}$$

Observation date was included as a random intercept to account for repeated scans within days. The harmonic terms were used as flexible basis functions to describe nonlinear variation across the observed daytime window and were not interpreted as evidence of a complete 24-hour or 12-hour behavioural cycle.

We restricted model fitting and population-level prediction to scans that were conducted between 05.00 and 18.00. Predicted values were back-transformed from the logit scale to probabilities. Internal peaks were identified as local maxima in the predicted curves and reported to the nearest 30 minutes. Uncertainty in the timing of a peak was estimated by repeatedly drawing the fixed-effect coefficients from their estimated multivariate normal sampling distribution and recalculating the predicted curve. Confidence intervals for local peak times were calculated conditional on a corresponding internal peak being detected in the simulated curve. Maxima occurring at 05.00 or 18.00 were classified as boundary maxima rather than precisely estimated internal peaks.

To examine seasonal patterns, we fitted the same harmonic models separately to dry- and wet-season observations and generated seasonal predicted curves and internal peak estimates. For the overall dataset and for each season separately, we tested the joint contribution of the four harmonic terms by comparing the full harmonic model with an intercept-only beta-binomial GLMM using a likelihood-ratio test. Because exploratory residual checks indicated dependence between temporally adjacent scans, we also conducted a sensitivity analysis in which alternating scans were retained within each observation day. We refitted the overall feeding and resting models to this temporally thinned dataset and compared coefficient directions, predicted curves, peak locations and joint harmonic tests with those from the full dataset.

## 3. Results

### 3.1. Daily and monthly climatic structure

Weather conditions during the study period varied strongly across days and months (figure 1, table 1). Across 353 weather-station monitoring days, the mean daily temperature was 17.1 ± 2.0°C, relative humidity 81.6 ± 11.6%, absolute humidity (WVP) 1.59 ± 0.20 kPa and wind speed 1.72 ± 1.41 m s^−1^. The mean daily daytime rainfall across all days was 2.25 ± 8.7 mm. On the 70 days that recorded rainfall, the mean daily daytime rainfall was 11.4 ± 16.7 mm. Temperature followed a consistent diel pattern across months, increasing from early morning to mid-afternoon, peaking between approximately 13.00 and 16.00, and then declining towards evening (figure 1A). WVP showed a broadly similar diel pattern, generally increasing from early morning to midday or mid-afternoon before declining later in the day, although the timing and magnitude of this pattern varied among months (figure 1C). The lowest daytime temperature recorded in any month was 8.3°C (July), while the highest daytime temperature reached 31.1°C in September. WVP varied widely across months, ranging from 0.6 kPa in September to 3.0 kPa in April (figure 1B,D). Wind speed also varied markedly among months, with monthly maximum daytime measurements ranging from 7.2 m s^−1^ in March–May to 13.3 m s^−1^ in November. Rainfall was highly episodic: all months included days with no daytime rainfall, whereas the maximum daily daytime totals reached 119.1 mm in April and 42.6 mm in March (table 1). Based separately on total 24-hour rainfall for each month, March, April, June, November, January and February were classified as wet months (greater than 100 mm), whereas May, July–October and December were classified as dry months (less than 100 mm).

**Figure 1.**
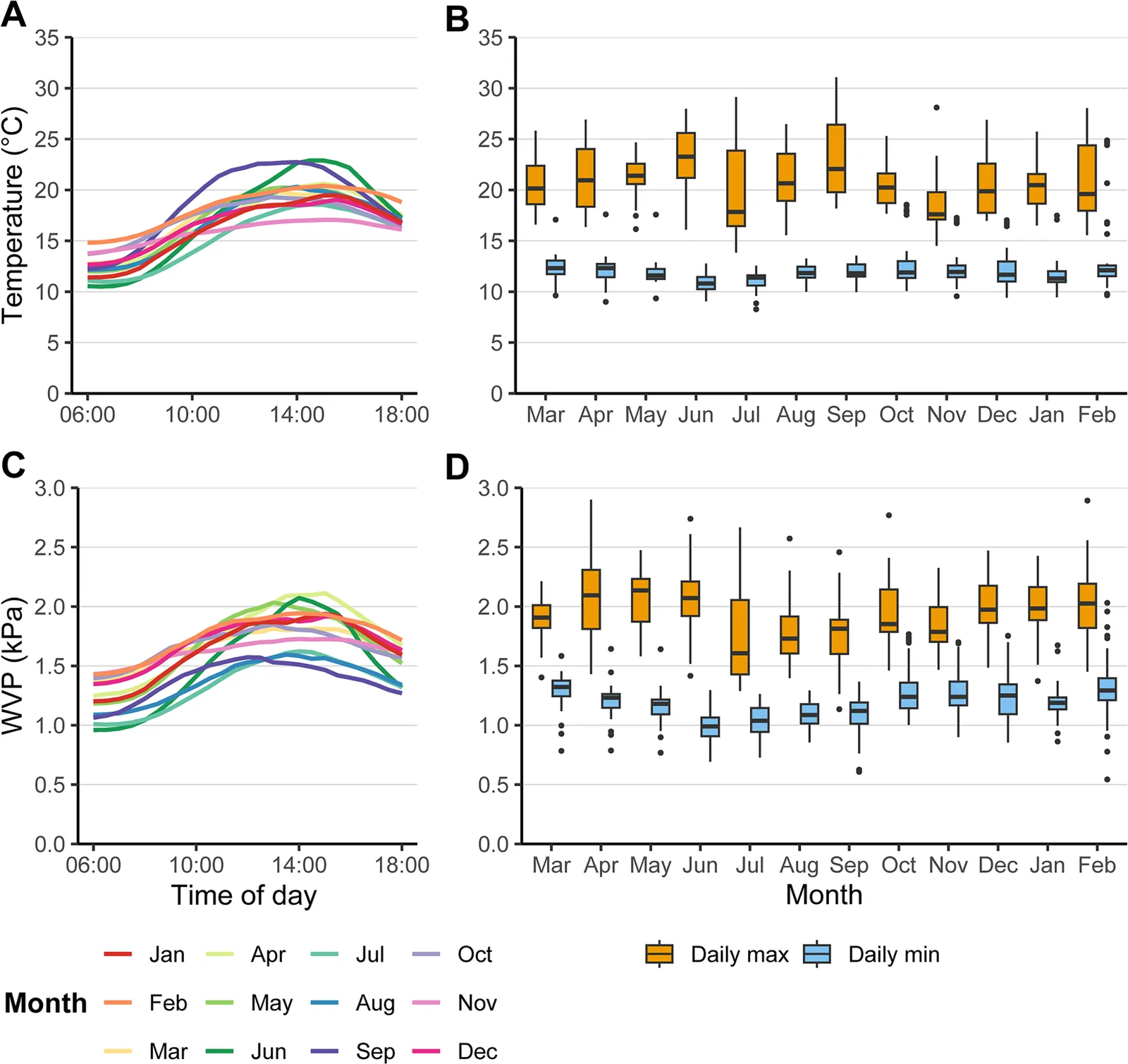
Daytime patterns and monthly distributions of daily maximum and minimum temperature and WVP, based on measurements recorded by the fixed weather station during the study period. Monthly daytime profiles of (A) temperature and (C) water vapour pressure (WVP) show average daytime patterns calculated from mean 30-minute values between 06.00 and 18.00. For (B) temperature and (D) WVP, daily maximum and minimum values were first calculated from daytime weather-station records between 06.00 and 18.00 for each date, and these daily values were then summarized by month using side-by-side box plots. In the box plots, the central line represents the median, the box indicates the interquartile range, and the whiskers show the range of values within 1.5 times the interquartile range. Outlying points, where present, represent values beyond this range.

**Table 1.** Monthly daytime climatic extremes, based on measurements recorded by the fixed weather station during the study period. Temperature, relative humidity (RH), water vapour pressure (WVP) and wind speed values represent the minimum and maximum individual measurements recorded between 06.00 and 18.00 within each month. Rainfall values represent the minimum and maximum daily daytime rainfall totals recorded during the same period.

month	temperature (°C)	RH (%)	WVP (kPa)	wind speed (m s^−1^**)**	rainfall (mm day^−1^**)**
Mar 2024	11.6–25.8	47.8–100.0	1.2–2.2	0.0–7.2	0.0–42.6
Apr 2024	10.8–26.9	69.4–100.0	1.1–3.0	0.0–7.2	0.0–119.1
May 2024	11.1–25.4	68.1–97.1	1.1–2.7	0.0–7.2	0.0–27.0
Jun 2024	9.1–28.0	52.1–93.8	0.8–2.7	0.0–8.6	0.0–28.2
Jul 2024	8.3–29.1	56.4–90.3	0.8–2.7	0.0–8.6	0.0–11.4
Aug 2024	9.0–26.3	42.0–95.6	0.7–2.0	0.0–9.7	0.0–0.0
Sep 2024	10.4–31.1	24.7–100.0	0.6–2.0	0.0–11.2	0.0–5.4
Oct 2024	9.9–26.6	41.0–100.0	0.9–2.4	0.0–12.2	0.0–13.5
Nov 2024	10.5–23.4	74.5–100.0	1.1–2.5	0.0–13.3	0.0–21.3
Dec 2024	9.4–24.9	67.1–100.0	1.0–2.8	0.0–12.2	0.0–8.1
Jan 2025	9.5–24.4	71.8–100.0	1.1–2.4	0.0–12.2	0.0–33.9
Feb 2025	10.4–28.1	42.7–100.0	1.0–2.9	0.0–12.2	0.0–19.5

### 3.2. The effect of season and climatic variables on activity

Across the study period, the chimpanzees devoted most observed activity to feeding (58.6%), followed by resting (27.5%), locomotion (7.7%) and grooming (6.3%). Fine-scale weather variables collectively improved the model fit for feeding (ΔAIC = −3.1; *χ*² = 11.07, d.f. = 4, *p* = 0.03), resting (ΔAIC = −5.5; *χ*² = 13.46, d.f. = 4, *p* = 0.009) and grooming (ΔAIC = −3.5; *χ*² = 11.56, d.f. = 4, *p* = 0.02), but not for locomotion (ΔAIC = −0.9; *χ*² = 8.86, d.f. = 4, *p* = 0.06). Across behaviours, marginal (*R*^2^) values ranged from 0.05 to 0.16, indicating modest explanatory power of the fixed effects, whereas conditional (*R*^2^) values ranged from 0.23 to 0.63, showing substantial additional variation among observation days.

Rainfall was associated with a redistribution of the activity budget away from feeding and towards resting. Feeding probability declined during rainfall (*β* = −0.48 ± 0.19, *χ*² = 6.68, d.f. = 1, *p* = 0.009; figure 2A; table 2), consistent with our prediction. Temperature, WVP and monthly fruit availability were not clearly associated with feeding, while there was weak evidence of a positive association between wind speed and feeding (*β* = 0.09 ± 0.05, *χ*² = 3.04, *p* = 0.08). Feeding varied across the day (*χ*² = 25.96, d.f. = 2, *p* < 0.001), with a higher probability in the afternoon than in the morning (*β* = 0.65 ± 0.16). Feeding probability was also higher in the wet season than in the dry season (*β* = 0.37 ± 0.17, *χ*² = 4.75, d.f. = 1, *p* = 0.02).

**Figure 2.**
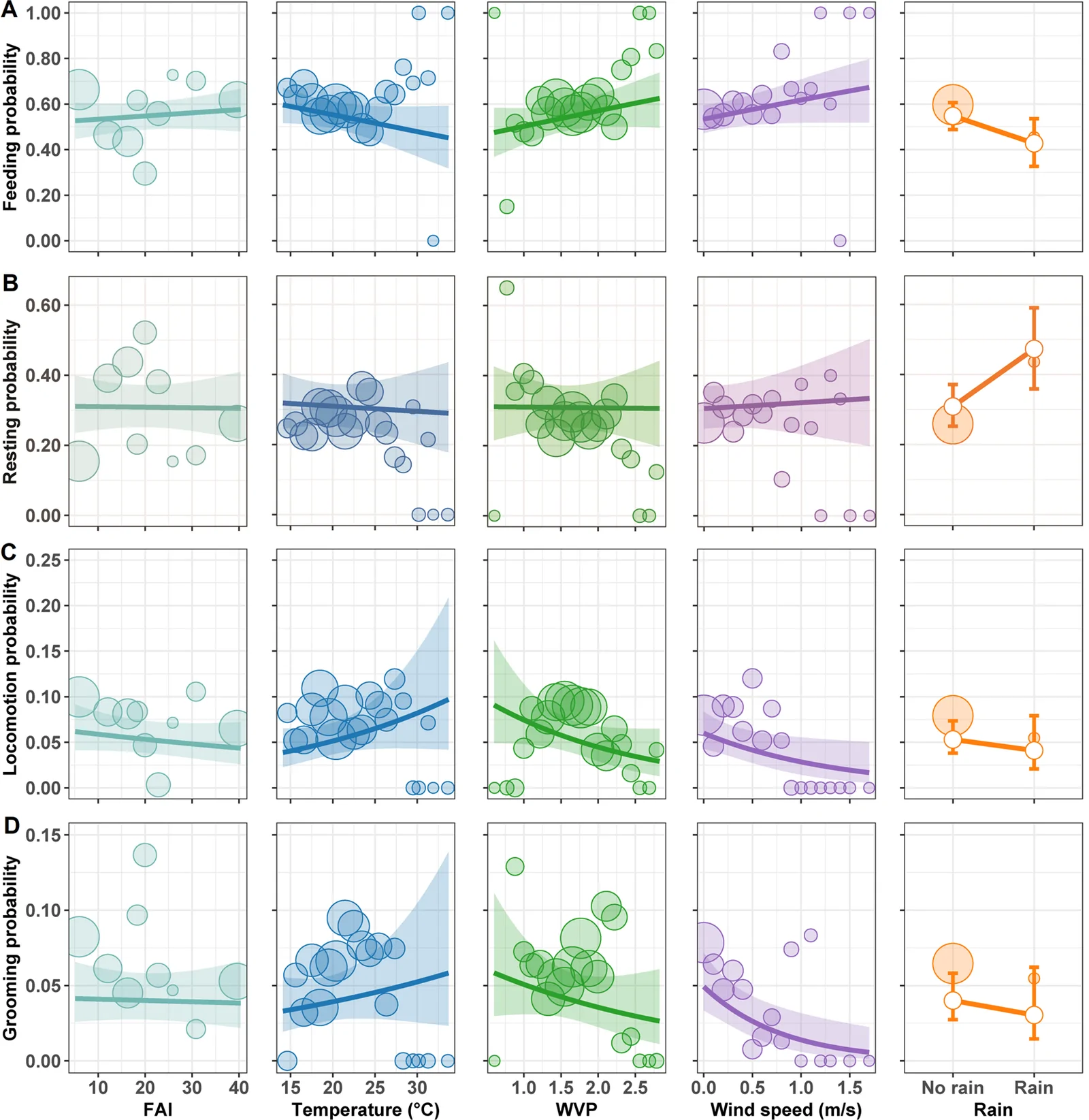
Effects of fruit availability and meteorological factors on chimpanzee activity probabilities. Predicted probabilities of four behavioural categories: (A) feeding, (B) resting, (C) locomotion and (D) grooming. Within each row, panels show relationships with fruit availability index (FAI), temperature (°C), water vapour pressure (WVP), wind speed (m s^−1^) and rainfall (0 = no rain, 1 = rain). Solid lines indicate fitted probabilities from beta-binomial GLMMs, with shaded bands showing 95% confidence intervals. Circles represent observed activity proportions within binned ranges of each continuous predictor, with circle size proportional to the number of scan observations per bin. Rainfall panels show predicted probabilities for no-rain and rain conditions with 95% confidence intervals.

**Table 2.** Results of beta-binomial GLMMs examining associations between meteorological variables, fruit availability, time of day, season and chimpanzee activity probabilities.

behaviour	predictor	estimate	s.e.	χ²	d.f.	*P*	marginal *R*²	conditional *R*²
feeding	intercept	0.19	0.12				0.16	0.63
	temperature	−0.09	0.06	2.41	1	0.12		
	WVP	0.09	0.06	2.05	1	0.15		
	wind speed	0.09	0.05	3.04	1	0.08		
	rain	−0.48	0.19	6.68	1	**0.009**		
	FAI	0.06	0.08	0.76	1	0.38		
	time of day			25.96	2	**<0.001**		
	midday	−0.17	0.11					
	afternoon	0.65	0.16					
	season (wet)	0.37	0.17	4.75	1	**0.02**		
resting	intercept	−0.81	0.14				0.09	0.47
	temperature	−0.02	0.06	0.11	1	0.74		
	WVP	−0.003	0.07	0.002	1	0.96		
	wind speed	0.02	0.05	0.13	1	0.71		
	rain	0.70	0.20	12.62	1	**<0.001**		
	FAI	−0.009	0.10	0.007	1	0.93		
	time of day			16.43	2	**<0.001**		
	midday	0.12	0.12					
	afternoon	−0.60	0.18					
	season (wet)	−0.55	0.20	7.53	1	**0.006**		
locomotion	intercept	−2.88	0.18				0.05	0.23
	temperature	0.15	0.10	2.36	1	0.12		
	WVP	−0.18	0.10	3.14	1	0.08		
	wind speed	−0.19	0.09	4.91	1	**0.03**		
	rain	−0.27	0.32	0.71	1	0.40		
	FAI	−0.12	0.11	1.19	1	0.28		
	time of day			1.60	2	0.45		
	midday	−0.23	0.18					
	afternoon	−0.13	0.25					
	season (wet)	0.41	0.22	3.51	1	0.06		
grooming	intercept	−3.18	0.20				0.08	0.25
	temperature	0.09	0.10	0.85	1	0.35		
	WVP	−0.12	0.10	1.41	1	0.24		
	wind speed	−0.33	0.11	9.05	1	**0.003**		
	rain	−0.29	0.33	0.76	1	0.38		
	FAI	−0.03	0.11	0.05	1	0.83		
	time of day			13.59	2	**0.001**		
	midday	0.45	0.19					
	afternoon	−0.76	0.37					
	season (wet)	0.14	0.24	0.35	1	0.55		

Resting probability increased during rainfall (*β* = 0.70 ± 0.20, *χ*² = 12.62, d.f. = 1, *p* < 0.001; figure 2B; table 2). Temperature, WVP, wind speed and fruit availability were not clearly associated with resting. Resting varied across the day (*χ*² = 16.43, d.f. = 2, *p* < 0.001), with a lower probability in the afternoon than in the morning (*β* = −0.60 ± 0.18). After accounting for immediate rainfall and the other predictors, resting probability was lower in the wet season than in the dry season (*β* = −0.55 ± 0.20, *χ*² = 7.53, d.f. = 1, *p* = 0.006).

Locomotion probability declined as wind speed increased (*β* = −0.19 ± 0.09, *χ*² = 4.91, d.f. = 1, *p* = 0.03; figure 2C; table 2), consistent with our prediction. Temperature, rainfall and fruit availability were not clearly associated with locomotion, while there was weak evidence of a negative association for WVP (*β* = −0.18 ± 0.10, *χ*² = 3.14, *p* = 0.08). Locomotion did not vary clearly across the day (table 2), and there was weak evidence that locomotion probability was higher in the wet season than in the dry season (*β* = 0.41 ± 0.22, *χ*² = 3.51, *p* = 0.06).

Wind speed was the only climatic variable that was clearly associated with grooming. Grooming probability declined as wind speed increased (*β* = −0.33 ± 0.11, *χ*² = 9.05, d.f. = 1, *p* = 0.003; figure 2D; table 2). Temperature, WVP, rainfall, fruit availability and season were not clearly associated with grooming. Grooming varied across the day (χ² = 13.59, d.f. = 2, p = 0.001), with a higher probability at midday than in the morning (*β* = 0.45 ± 0.19) and a lower probability in the afternoon than in the morning (*β* = −0.76 ± 0.37).

### 3.3. Diel rhythms in feeding and resting

The fitted harmonic models indicated clear daytime variation in feeding and resting across the annual dataset ( figure 3A), consistent with our prediction. Feeding showed internal maxima at approximately 07.00 (95% CI: 06.00–07.00) and 16.00 (95% CI: 15.30–17.00), with predicted probabilities of 0.74 and 0.75, respectively. In the overall feeding model, cos1 (*β* = −2.45, *p* = 0.002), sin2 (*β* = 0.56, *p* = 0.004) and cos2 (*β* = −1.57, *p* < 0.001) were significant, whereas sin1 was not (*β* = 0.40, *p* = 0.103). Resting showed an approximately complementary pattern, with an internal maximum at approximately 11.00 (95% CI: 11.00–11.30) and a predicted probability of 0.38. For resting, cos1 (*β* = 2.54, *p* = 0.003), sin2 (*β* = −0.63, *p* = 0.004) and cos2 (*β* = 1.52, *p* < 0.001) were significant, whereas sin1 was not (*β* = −0.45, *p* = 0.097). These patterns were robust to temporal thinning. After retaining alternating scans within each observation day, coefficient directions remained consistent, and the joint harmonic effects remained significant for both feeding (*χ*² = 44.3, d.f. = 4, *p* < 0.001) and resting (*χ*² = 34.1, d.f. = 4, *p* < 0.001).

**Figure 3.**
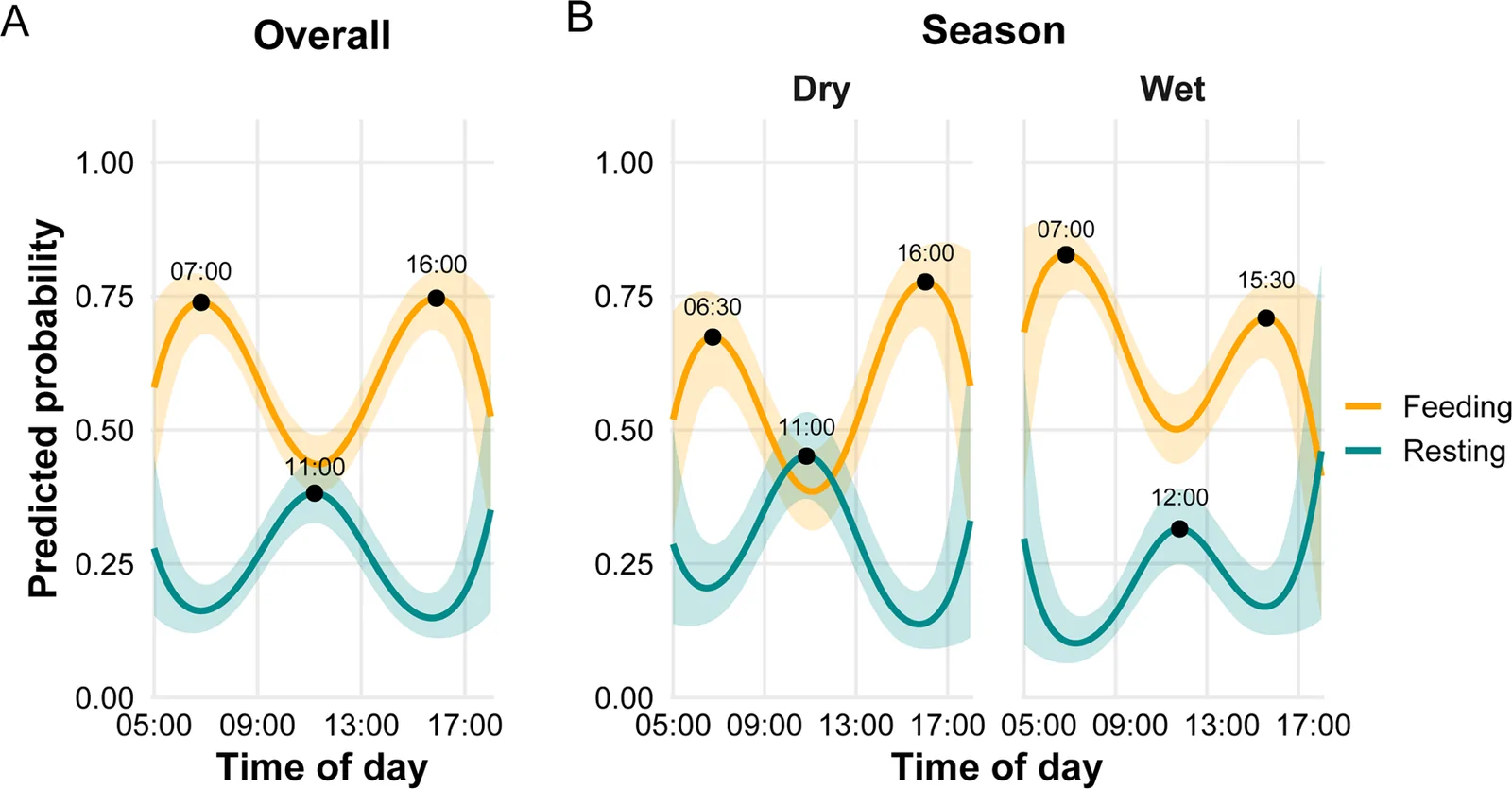
Diel variation in the probability of feeding and resting estimated using harmonic beta-binomial GLMMs. Predicted probabilities of feeding and resting across the day. (A) Population-level diel patterns. (B) Diel patterns under dry and wet conditions. Lines show fitted harmonic-model predictions, and shaded bands indicate 95% confidence intervals. Black points and labels mark the timing of predicted daily peaks.

Season-specific models showed broadly similar patterns, but there was some descriptive seasonal variation (figure 3B; table 3). In the dry season, feeding peaked at approximately 06.30 (95% CI: 06.00–07.30) and 16.00 (95% CI: 15.30–17.00), with predicted probabilities of 0.67 and 0.78, respectively; cos1, sin2 and cos2 were significant, whereas sin1 was not. Resting showed an internal maximum at approximately 11.00 (95% CI: 10.30–11.00), with a predicted probability of 0.45; cos1, sin2 and cos2 were significant, whereas sin1 was not (table 3). In the wet season, feeding peaked at approximately 07.00 (95% CI: 06.00–07.30) and 15.30 (95% CI: 15.00–17.00), with predicted probabilities of 0.83 and 0.71, respectively, and all four harmonic terms were individually significant. Resting peaked at approximately 12.00, with a predicted probability of 0.32; cos1 (*β* = 3.51, *p* = 0.012) and cos2 (*β* = 1.85, *p* < 0.001) were significant, whereas sin1 (*β* = −0.82, *p* = 0.053) and sin2 (*β* = −0.51, *p* = 0.119) were not. Nevertheless, the four harmonic terms were jointly significant for feeding and resting in both seasons.

**Table 3.** Diel harmonic model coefficients for feeding and resting behaviour.

model	coefficient	estimate	s.e.	*z*	*p*-value	s.d. (date)	dispersion
feeding–overall	Intercept	−1.07	0.528	−2.03	0.042	0.742	0.901
	sin1	0.399	0.244	1.63	**0.103**		
	cos1	−2.45	0.782	−3.13	**0.002**		
	sin2	0.557	0.196	2.84	**0.004**		
	cos2	−1.57	0.292	−5.38	**<0.001**		
resting–overall	Intercept	0.453	0.582	0.78	0.436	0.877	0.939
	sin1	−0.448	0.270	−1.66	**0.097**		
	cos1	2.54	0.862	2.94	**0.003**		
	sin2	−0.628	0.215	−2.91	**0.004**		
	cos2	1.52	0.321	4.73	**<0.001**		
feeding–dry season	Intercept	−1.11	0.697	−1.60	0.110	0.862	0.703
	sin1	0.140	0.330	0.42	0.672		
	cos1	−2.36	1.03	−2.28	**0.023**		
	sin2	0.587	0.268	2.19	**0.029**		
	cos2	−1.59	0.390	−4.07	**<0.001**		
resting–dry season	Intercept	0.430	0.740	0.58	0.561	0.907	0.852
	sin1	−0.305	0.351	−0.87	0.385		
	cos1	2.26	1.10	2.06	**0.039**		
	sin2	−0.774	0.284	−2.73	**0.006**		
	cos2	1.44	0.409	3.52	**<0.001**		
feeding–wet season	Intercept	−1.09	0.828	−1.31	0.189	0.477	1.35
	sin1	0.880	0.361	2.44	**0.015**		
	cos1	−2.73	1.23	−2.22	**0.027**		
	sin2	0.616	0.283	2.18	**0.029**		
	cos2	−1.62	0.453	−3.57	**<0.001**		
resting–wet season	Intercept	0.878	0.940	0.93	0.350	0.707	1.24
	sin1	−0.816	0.422	−1.94	**0.053**		
	cos1	3.51	1.40	2.51	**0.012**		
	sin2	−0.512	0.328	−1.56	**0.119**		
	cos2	1.85	0.519	3.57	**<0.001**		

## 4. Discussion

Our year-long study showed that Mayebe chimpanzees maintained pronounced daytime activity rhythms while selectively adjusting behavioural allocation in relation to local meteorological conditions. Rainfall and wind speed showed the clearest behavioural association, whereas temperature, WVP and monthly fruit availability showed no consistent relationship with activity. These findings suggest that immediate weather conditions influence particular components of the activity budget without substantially altering the broader daily organization of feeding and resting.

Among the meteorological variables examined, temperature and WVP showed no clear or consistent association with chimpanzee activity. This was unexpected because some previous work has identified that thermal and atmospheric moisture conditions can shape primate activity across both hot and cold environments [2,34,37,66,100–103]. In NNP, both temperature and WVP increased from the cool early morning to maxima around midday or early afternoon before declining towards evening. Resting also varied strongly across the day, with a late-morning peak, but neither temperature nor WVP showed a clear independent association with resting after accounting for time of day and other covariates. Humidity can affect behaviour by impeding evaporative heat loss and increasing the thermal costs of activity, particularly under warm conditions [20]. For example, wild Barbary macaques spent less time socializing at higher relative humidity, although humidity was not clearly associated with moving or resting [40]. Our findings also differ from those reported in hotter savannah and open-woodland environments, where elevated temperatures can suppress locomotion and increase resting in chimpanzees [52,54]. Primates in colder environments may also adjust their activity in response to temperature. Barbary macaques and black-and-white snub-nosed monkeys increase feeding or foraging under colder conditions, while black-and-white snub-nosed monkeys also spend less time resting during winter [29,40]. The absence of clear temperature and WVP effects in Nyungwe may therefore indicate that the conditions encountered remained within ranges that did not impose sufficiently strong thermal or moisture-related constraints to alter activity allocation.

Our prediction that chimpanzees would decrease feeding and increase resting under rainy conditions was supported. Rainfall emerged as one of the strongest meteorological predictors of behavioural variation: feeding probability declined during rainfall events, whereas resting increased. This pattern suggests that precipitation imposed immediate constraints on feeding and promoted a shift towards inactivity. Rainfall can reduce visibility, increase the energetic costs of movement on wet substrates and limit access to arboreal feeding sites, making foraging less efficient or less favourable during rainfall. Under such conditions, a temporary reduction in feeding and increase in resting may represent an energetically efficient strategy until conditions improve. Similar responses to rainfall have been reported in mountain gorillas, where precipitation is associated with reduced activity and increased resting behaviour [104]. Comparable patterns have also been documented in other primates, where rainfall reduces feeding activity and promotes inactivity or sheltering behaviour [40,67,105].

Wind speed had its clearest negative associations with locomotion and grooming, both of which declined as wind speed increased within the observed range. Wind exposure may constrain arboreal movement by increasing energetic costs and reducing branch stability, thereby increasing the difficulty and risk of travelling or maintaining balance during grooming in exposed canopy positions. A comparable decline in grooming with increasing wind speed has been reported for chacma baboons in South Africa [2]. However, scan-level wind speeds were generally low, with no detectable wind during 59% of scans, and measurements taken from nearby elevated positions were only a proxy for canopy-level exposure. Moreover, because we did not distinguish whether locomotion and grooming occurred on the ground or in trees, we could not determine whether these associations were driven primarily by wind exposure in the canopy. The wind-related findings should therefore be interpreted as associations within the limited range of conditions observed.

Feeding probability was higher, and resting probability lower, during the wet season, suggesting a seasonal redistribution of activity towards feeding. Thus, the immediate response to an active rainfall event differed from the broader association with wet-season conditions. This resembles the reduced rainy-season resting reported for Assamese macaques [106], but contrasts with grey-shanked douc langurs, which fed and travelled more during the dry season [107], and Taï chimpanzees, which increased feeding during the minor dry season when preferred foods were less available [62]. In Nyungwe, fruit availability has been reported to decline during wetter periods and peak during the major dry season [79]. The greater wet-season feeding observed at Mayebe may, therefore, reflect increased feeding effort when fruit resources are less abundant, although monthly fruit availability was not independently associated with feeding in our models. These contrasting patterns suggest that seasonal activity responses depend on local resource conditions and energetic demands rather than rainfall season alone.

Many primates, including chimpanzees, show a bimodal feeding pattern, with peaks in the morning and late afternoon with rest during the middle of the day [54,62,108–110]. Our harmonic beta-binomial GLMM revealed a similar pattern in Mayebe chimpanzees. Such diel rhythms are commonly interpreted as behavioural strategies that balance energetic demands with thermoregulatory constraints, with individuals reducing energetically costly activities during periods of less favourable thermal conditions [54,77,107,111]. Although chimpanzees showed a broadly similar bimodal feeding rhythm in both seasons, the predicted timing and height of the peaks differed slightly. In the wet season, the morning feeding peak occurred slightly later and the afternoon peak slightly earlier than in the dry season, suggesting a modest seasonal compression of feeding activity within the day. The higher morning but lower afternoon feeding probability in the wet season may indicate that chimpanzees shifted feeding effort towards the earlier part of the day under wetter conditions. The slight seasonal shifts may reflect differences in nest-departure timing, food distribution or accessibility, although these mechanisms were not directly tested. Resting retained an approximately complementary midday pattern in both seasons, but its peak occurred slightly later and was lower in the wet season, further suggesting seasonal flexibility in the timing and allocation of daily activities. Together, the results suggest that Mayebe chimpanzees maintained a broadly conserved daily rhythm across seasons while showing limited flexibility in the timing of feeding and resting. Similar seasonal variation in primate activity schedules has been linked to meteorological conditions and food availability [2,77,105].

Overall, our findings indicate that chimpanzees in Nyungwe's cool montane forest maintain relatively stable daily activity rhythms across seasons while adjusting specific components of their activity budgets in response to fine-scale meteorological variation. Although the general bimodal feeding rhythm resembled patterns reported for other chimpanzee populations, the strength and direction of several meteorological associations differed from those documented in hotter or more open habitats. In particular, the limited influence of temperature and WVP, together with clear behavioural responses to rainfall and wind speed, suggests that temperature and atmospheric moisture were less constraining than rainfall and wind within the conditions observed. However, the interpretation of these findings is subject to several methodological considerations. Individual identity could not be incorporated into the scan-level models, and microclimatic measurements taken at chimpanzee-selected locations may reflect both environmental exposure and microhabitat choice. Moreover, the mutually exclusive behaviours were analysed using separate one-versus-rest models, wind measurements provided only an approximation of canopy-level exposure, and the harmonic analyses were restricted to the observed 05.00–18.00 daytime window. Despite these limitations, the findings underscore the importance of considering fine-scale meteorological variation when interpreting primate behavioural ecology. The ability of chimpanzees to modify feeding, resting, locomotion and grooming in response to short-term changes in rainfall, wind and other local conditions may provide an important behavioural mechanism for buffering climatic variability without substantially altering their overall daily activity rhythm. Such behavioural flexibility could therefore contribute to the capacity of chimpanzees to cope with some aspects of ongoing climate change, although its effectiveness will depend on whether suitable microhabitats, food resources and structurally diverse habitats remain available. Understanding both the scope and limits of this flexibility will be important for predicting the resilience of chimpanzee populations under future climatic conditions.

## Data Availability

The data and R codes are provided on Figshare [112].
